# Sarcopenic Obesity: An Emerging Public Health Problem, But an Answer Appears to Be Available

**DOI:** 10.14336/AD.2021.1118

**Published:** 2022-06-01

**Authors:** Marco Machado

**Affiliations:** Universidade Iguaçu Campus V, Itaperuna, RJ, Brazil

**To the editor,**

Recently Tong Ji et al [1] published a work of great importance for public health. The problems associated with aging will increase over the next 3 decades following the growth rate in the age group above 60 years old (wich will be about 21% in 2050). One of these problems is sarcopenic obesity (SO), which is a loss of muscle mass, strength and increase in body fat. SO causes frailty, falls, disability, immobility, fractures, cardiometabolic and respiratory diseases, cancer, and increased mortality.

After showing the different methodologies for diagnosis and the physiological mechanisms of SO, Tong Ji et al [1] discuss intervention and treatment proposals such as physical exercise and dietary intervention strategies, as would be expected in the topic. However, I would like to add contribution to the excellent work of the authors.

Among the nutritional strategies against SO in the elderly, creatine supplementation has been proposed as the most effective when associated with physical exercise [2-4]. Chilibeck et al [4] show in a meta-analysis (22 studies; n=722s) significant gain in lean mass (mean difference =1.44 [95% CI =1.02-1.86] kg; p<0.00001) among people aged 57 to 70 years supplemented with creatine plus exercise compared to placebo. Additionally, they showed that creatine supplementation increases strength in upper and lower limbs.

About frailty and risk of falls, Candow et al [6] showed that elderly individuals performing resistance exercises plus creatine supplementation improved the efficiency in the sit-to-stand test by 23%, while those who performed only the exercise improved by 16%. The sit-and-stand test is recognized as one of the best tests to assess the risk of falls. Devries & Phillips [5] showed that creatine supplementation, as compared with placebo, during RT significantly increased the number of chair stands completed in 30 s (weighted mean difference, 1.93 stands; 95% CI, 0.19 to 3.67 stands; P = 0.03, ES, 1.19). These meta-analyses strengthen the empirical evidence for the adoption of creatine supplementation as an enhancer of the functional capacity of the elderly in facing frailty and the risk of falls.

Creatine supplementation contributes to combat SO through several mechanisms, the increase in the intramuscular CrP concentration (with strong empirical evidence) allows more efficiency during exercise, enhancing its effects [3]. Other mechanisms still lack more empirical evidence but seem to involve endocrine, immunological and biomolecular factors (activation of the AKT-mTOR pathway and inhibition of myostatin)[3,4].

I could not fail to mention briefly, in order not to escape the subject, that studies show effects of creatine supplementation on bone health and cognition in elderly people [6]. Increased memory retention, reaction time and intelligence tests’ results have been observed by several researchers around the world [7-9]. The findings indirectly affect the reduction of frailty and the risk of falls, in addition to contributing to the quality of life of the elderly.

Last but not least is to highlight the accumulation of evidence that creatine supplementation is safe for the elderly’s health [2,10]. It has been increasingly shown that kidney problems, thermoregulatory complications and cramps are rare or even non-existent when creatine supplementation is used at recommended doses [10]. It is important to emphasize that there is a risk of contaminated supplements, regulatory agencies tend to be less strict with nutritional supplements compared to drugs.

There are still limitations on the relationship of creatine supplementation and longevity. For this, studies with longer follow-up and with a large number of participants would be necessary. However, there is a consensus that the elderly are greatly benefited by the adoption of creatine supplementation added to physical exercise.

I conclude by congratulating the editor and the authors [1] for their elegant work and hope to have added elements to broaden the discussion of the topic.
